# Effects of biochar types on seed germination, growth, chlorophyll contents, grain yield, sodium, and potassium uptake by wheat (*Triticum aestivum* L.) under salt stress

**DOI:** 10.1186/s12870-024-05188-0

**Published:** 2024-06-01

**Authors:** Sumei Duan, Arwa Abdulkreem AL-Huqail, Ibtisam Mohammed Alsudays, Mobeen Younas, Alishba Aslam, Ahmad Naeem Shahzad, Muhammad Farooq Qayyum, Muhammad Rizwan, Yousef Alhaj Hamoud, Hiba Shaghaleh, Jean Wan Hong Yong

**Affiliations:** 1 College of Agriculture, Anhui Science and Technology University, Chuzhou City, 233100 Anhui China; 2 Department of Biology, College of Science, Princess Nourah bint Abdulrahman University, P.O.Box 84428, Riyadh, 11671 Saudi Arabia; 3 Department of Biology, College of Science, Qassim University, Burydah, 52571 Saudi Arabia; 4 Department of Soil Science, Faculty of Agricultural Sciences & Technology, Bahauddin Zakariya University Multan, Multan, 60800 Pakistan; 5 Institute of Agronomy, Faculty of Agricultural Sciences & Technology, Bahauddin Zakariya University Multan, Multan, 60800 Pakistan; 6 Department of Environmental Sciences, Government College University Faisalabad, Faisalabad, 38000 Pakistan; 7 College of Hydrology and Water Resources, Hohai University, Nanjing, 210098 China; 8 College of Environment, Hohai University, Nanjing, 210098 China; 9 Department of Biosystems and Technology, Swedish University of Agricultural Sciences, Alnarp, 23456 Sweden

**Keywords:** Salinity, Cereals, Pyrolysis, Organic-waste-management

## Abstract

Soil salinity is a significant challenge in agriculture, particularly in arid and semi-arid regions such as Pakistan, leading to soil degradation and reduced crop yields. The present study assessed the impact of different salinity levels (0, 25, and 50 mmol NaCl) and biochar treatments (control, wheat-straw biochar, rice-husk biochar, and sawdust biochar applied @ 1% w/w) on the germination and growth performance of wheat. Two experiments: a germination study and a pot experiment (grown up to maturity), were performed. The results showed that NaCl-stress negatively impacted the germination parameters, grain, and straw yield, and agronomic and soil parameters. Biochar treatments restored these parameters compared to control (no biochar), but the effects were inconsistent across NaCl levels. Among the different biochars, wheat-straw biochar performed better than rice-husk and sawdust-derived biochar regarding germination and agronomic parameters. Biochar application notably increased soil pH_s_ and electrical conductivity (EC_e_). Imposing NaCl stress reduced K concentrations in the wheat shoot and grains with concomitant higher Na concentrations in both parts. Parameters like foliar chlorophyll content (a, b, and total), stomatal and sub-stomatal conductance, and transpiration rate were also positively influenced by biochar addition. The study confirmed that biochar, particularly wheat-straw biochar, effectively mitigated the adverse effects of soil salinity, enhancing both soil quality and wheat growth. The study highlighted that biochar application can minimize the negative effects of salinity stress on wheat. Specifically, the types and dosages of biochar have to be optimized for different salinity levels under field conditions.

**Supplementary Information:**

The online version contains supplementary material available at 10.1186/s12870-024-05188-0.

## Introduction

Salinity (the excess of soluble salts in a growth medium) is a significant abiotic stress that could harm the germination, growth, and yield of plants [1] by limiting water availability and causing ion toxicity [2]. In soils, salinity may arise due to weathering of soil parent material (primary salinity) and agricultural practices such as irrigation, agricultural intensification, and excessive mineral fertilization [3–5]. Salt-affected soils are a major global issue agriculture. According to an estimate, more than 1100 million hectares of agricultural land are salt-affected worldwide [6]. Among these, 60% are saline, 26% are sodic, and 14% are saline-sodic. The vital metabolic processes of plants are affected by soil salinity and thus affecting physiology, lowering growth, reduced quality and yield attributes [7–10]. Additionally, salt-affected soils become degraded and lose their ability to produce good-quality crops and other necessary ecosystem functions.

Several techniques, such as using salt-tolerant crop varieties and improving irrigation efficiency, fertilization, and organic amendments, have been used to minimize the losses associated with salinity [11, 12]. Good quality irrigation water and management practices may also contribute to obtaining optimum yield [13]. However, such practices are less cost-effective and time-consuming. Therefore, farmers prefer to use certain amendments to mitigate salinity effects. These amendments, either organic (farm manure, crop residues, press mud, etc.) or inorganic (gypsum, sulfur, etc.), may enhance soil fertility and productivity through increasing permeability, leaching of salts, biostimulants, and solubilizing carbonates [12, 14, 15].

Using various biochars (BCs) as amendments have recently become famous for enhancing crop productivity in salt-affected soils [16, 17]. BC, including nano-biochar, is a carbon-rich organic material prepared through pyrolysis of organic waste materials [18, 19]. Interestingly, after the addition to soils, BC may improve soil physical properties, water-holding capacities, and mitigate greenhouse gases through enhanced carbon sequestration [18, 20–22]. Apart from the heterocyclic carbon compounds, BC may also contain heterogeneous organic (stable and unstable) components, ash (minerals), and volatile matter in various proportions depending on the feedstock type and pyrolysis conditions [23].

Most studies reported the addition of BC in either standard or degraded soils [12, 24–26]. The effects of adding BC is unclear in saline soils because of the variability of source materials to produce BC and especially those with high minerals and ash contents. Interestingly, some studies reported salinity mitigation and improved crop performance in response to BC additions. For instance, Thomas et al. reported that surface application of 50 tons BC per ha mitigated salinity through the sorption of salts, and enhanced plant biomass and physiological attributes [27]. Lashari et al. also reported mitigation of sodicity with the application of co-compost of BC- manure-compost and described this due to adsorption of Na [28]. Hammer et al. also said that BC mitigated the salinity-induced adverse effects on plant growth through ion adsorption [29]. Huang et al. reported that applying wood-derived BC (prepared at 600 °C) in an acidic soil alleviated the negative impact of salt stress on rice, modified soil properties, and regulated the bacterial abundance in soil [30]. In another study, El-Sharkawy et al. used acid-modified BCs (prepared using rice straw and cotton stalks) in a saline-sodic soil [31]. They investigated the influence of BCs on soil properties, nutrient dynamics, and crop productivity under maize-wheat cultivation. They reported that acids modified the porosity, functional groups, and water-holding capacity of the BCs, which were responsible for mitigating salinity stress. As discussed earlier, the positive influence of BCs on plant growth under saline or saline-sodic conditions, the mechanisms underlying the effects of various BC types (varying in ash content and functional groups) on saline and alkaline-calcareous soils, are unclear.

In the present study, wheat (*Triticum aestivum* L.) was grown as a test crop, as it is one of the most important cereal crops used as a staple food [32]. In Pakistan, wheat is a major cereal crop grown on about 9.0 million hectares of land [33] and its productivity is affected by salinity, which reduces photosynthesis, plant growth, and development [34, 35]. The aims of the study were to evaluate the effects of salt stress and different biochar types on (1) seed germination of wheat; (2) growth, gas exchange characteristics and foliar chlorophyll contents; (3) Na and K uptake by wheat; (4) various soil chemical properties.

## Materials and methods

Two parallel experiments (a seed germination trial and a pot experiment) were conducted under ambient conditions. The climate of the experimental location was arid subtropical continental (mean air temperature (maximum and minimum during cropping period) ranged from 44 °C (23 °C) in May to 22 °C (13 °C) in January, and 25 mm rainfall during crop period). The biochars were prepared using different feedstock such as wheat straw, rice husk, and sawdust. The collected feedstock was sun-dried, and pyrolysis was done. The pyrolysis of wheat straw was done in a specially designed kon-tiki biochar furnace [36]. In this furnace, flame curtain pyrolysis technique is involved. The feedstock is added in the furnace layer by layer. The burning/combustion starts from the bottom layer to above layers. In this technique, overall emission of greenhouse gases is lower than traditional methods of biochar/charcoal production. However, the exact temperature and time cannot be calculated because it may greatly vary depending on feedstock type. The pyrolysis of rice husk and sawdust were done in vertical silo type reactor (a closed stainless-steel, container which is heated through gas supplied burner) following Qayyum et al. [37]. The prepared BCs were stored for analyses and experimental purposes.

For both trials, the main factor was biochar treatments, i.e., control and three biochars (wheat straw biochar (WSB), rice husk biochar (RHB), and sawdust biochar (SDB). The second factor was salinity (different concentrations of NaCl solution, i.e., 0 mmol NaCl, 25 mmol NaCl, and 50 mmol NaCl). An approved variety of wheat (AS-202) was selected for both experiments. The detailed characteristics of BCs are given in supplementary file as Table S1 [38]. The soil used for the present experiment was fine silty, mixed, hyperthermic Sodic Haplocambid (according to USDA classification), an aridisol, well-drained, alkaline (pH_s_ 7.8), EC_e_ 0.85 dSm^− 1^, OM 0.5%, weakly structured, and moderately to strongly calcareous (CaCO_3_ > 10%) in nature [38].

### Seed germination trial

Washed sand was filled in the plastic trays (2 kg sand in each tray) and the biochar treatments were applied in respective trays. A specific amount of water was used in each tray to get a favorable moisture content for wheat germination. In the trays, getting salinity treatment, NaCl solutions were applied for irrigation. Fifty seeds of wheat were sown in each tray. The germination of the seedlings was noted, and different parameters such as germination percentage, coefficient of uniformity of emergence, and mean emergence time were calculated using collected data.

### Pot trial

For the pot experiment, plastic pots of a capacity of 10 kg were used for plant growth. The biochar treatments (50 g per pot or 1% w/w) were mixed with 5 kg soil (air-dried and sieved through 2 mm collected from the experimental location described above), and mixtures were filled into experimental pots. Each treatment was replicated four times. In control pots, only soil was added. Eight wheat seeds were sown in each pot at equal distances and irrigated with tap water to attain 60% WHC, maintained throughout the experiment by weighing the pots. However, after germination, NaCl solutions (0 mM NaCl, 25 mM NaCl, and 50 mM NaCl) were applied in salinity-treatment pots as irrigation water. After 3–4 weeks od germination, fertilizers (N-P-K, using urea, DAP, and SOP) were applied in all pots to get better results in the growth and yield of plants.

The plants were grown to maturity and harvested on 15th April 2019, following 19 weeks of germination. Different data, such as the number of spikes, spike length, shoot length, and biomass, were calculated, and samples were stored in plastic bags.

### Soil and plant analyses

Soil samples were collected from the pots, dried under sunlight, and in a hot air oven at 65 °C, then passed through a 2 mm sieve. The spikes from the wheat plants were carefully removed, and the seeds were extracted from each spike and placed in paper bags. The shoots were crushed using a grinding machine, and the materials were preserved in bags for further analyses.

Soil samples were analyzed for different characteristics such as soil pH_s_, soil EC_e_ extractable potassium, and sodium before and after the experimentation following recommended procedures. Soil pH_s_ and EC_e_ were measured in saturated paste and extracts respectively using BANTE PHS and BANTE BDS, respectively. For extractable K and Na, 5 g soil was extracted with 33 mL of 1 N Ammonium acetate solution through shaking and filtration. The procedure was repeated three times, and the final volume of the filtrate was made up to 100 mL The readings of K and Na were taken using a Flame Photometer (Jenway PFP 07).

For the determination of potassium and sodium concentrations in different plant parts such as grains and straw, a 0.5 g ground sample was digested in 10 mL di-acid (nitric acid and perchloric acid in a 2:1 ratio) using block digestor till colorless solution formed. The volume of digestate was made up to 100 mL using distilled water and filtered through filter papers (Whatman filter 42). The filtrate was further diluted as per requirements. The readings of the K and Na were taken with a flame photometer (Jenway PFP 7.0).

The gas exchange properties gs (stomatal conductance, mol m^− 2^ s^− 1^), ci (sub stomatal CO_2_, µmol mol^− 1^), E (transpiration rate, mmol m^− 2^ s^− 1^) and A (CO_2_ assimilation rate (µmol m^-2^ s^-1^) of all plots (both experiments) were measured using a portable and open gas exchange system using Infrared Gas Analyser technology (LCi-SD, ADC BioScientific Ltd., United Kingdom) following Khalid et al. [39] and Yong et al. [40]; the gas exchange calculations were in accordance to Farquhar et al. [41].

### Statistical analysis

All statistical analyses were performed using Statistix 8.1. Two-way ANOVA and Tukey’s HSD were performed where required. The Pearson correlation and principal component analysis were performed using R.

**Table 1:** Analysis of variance table (F-values) for investigated parameters of the experiment

Source variable	Treatment	Salinity	Treatment*Salinity
Soil EC	24.38*	71.85*	6.62*
Soil pH	23.76*	44.52*	0.87ns
Grain Yield	8.54*	66.44*	0.80ns
Straw Yield	9.85*	121.00*	0.26ns
1000 Grains Weight	21.71*	156.19*	0.61ns
Plant Height	42.85*	395.57*	12.07*
Chlorophyll a	74.51*	3106.43*	75.79*
Chlorophyll b	4.62*	14.12*	52.72*
Carotenoids	21.99*	215.09*	6.09*
Carbon Dioxide Assimilation	91.15*	189.50*	1.68ns
Stomatal Conductance	6.39*	111.36*	0.58ns
Transpiration rate	76.45*	237.87*	2.24ns
Sub Stomatal CO2	39.59*	206.68*	2.07ns
Mean Emergence Time	3.43*	0.88ns	2.44*
Coefficient of Uniformity of Emergence	3.21*	7.99*	3.81*
Seed Germination	4.08*	83.08*	0.71ns
Number of Fertile Tillers	5.42*	36.27*	0.52ns
Potassium conc. in Shoot	5.41*	162.62*	0.38ns
Sodium conc. in Shoot	21.62*	391.57*	1.23ns
Amm. Acet. Ext. Potassium in Soil	29.88*	133.07*	1.81ns
Amm. Acet. Ext. Sodium in Soil	39.32*	60.06*	1.83ns
Sodium Conc. in Grains	15.56*	92.70*	1.99ns
Potassium Conc. in Grains	18.00*	81.75*	0.32ns

* *P* ≤ 0.05, ** *P* ≤ 0.01, ***P ≤ 0.001, ns *P* > 0.05

## Results

### Effects of BCs on seed germination under salt stress

**Fig. 1:**
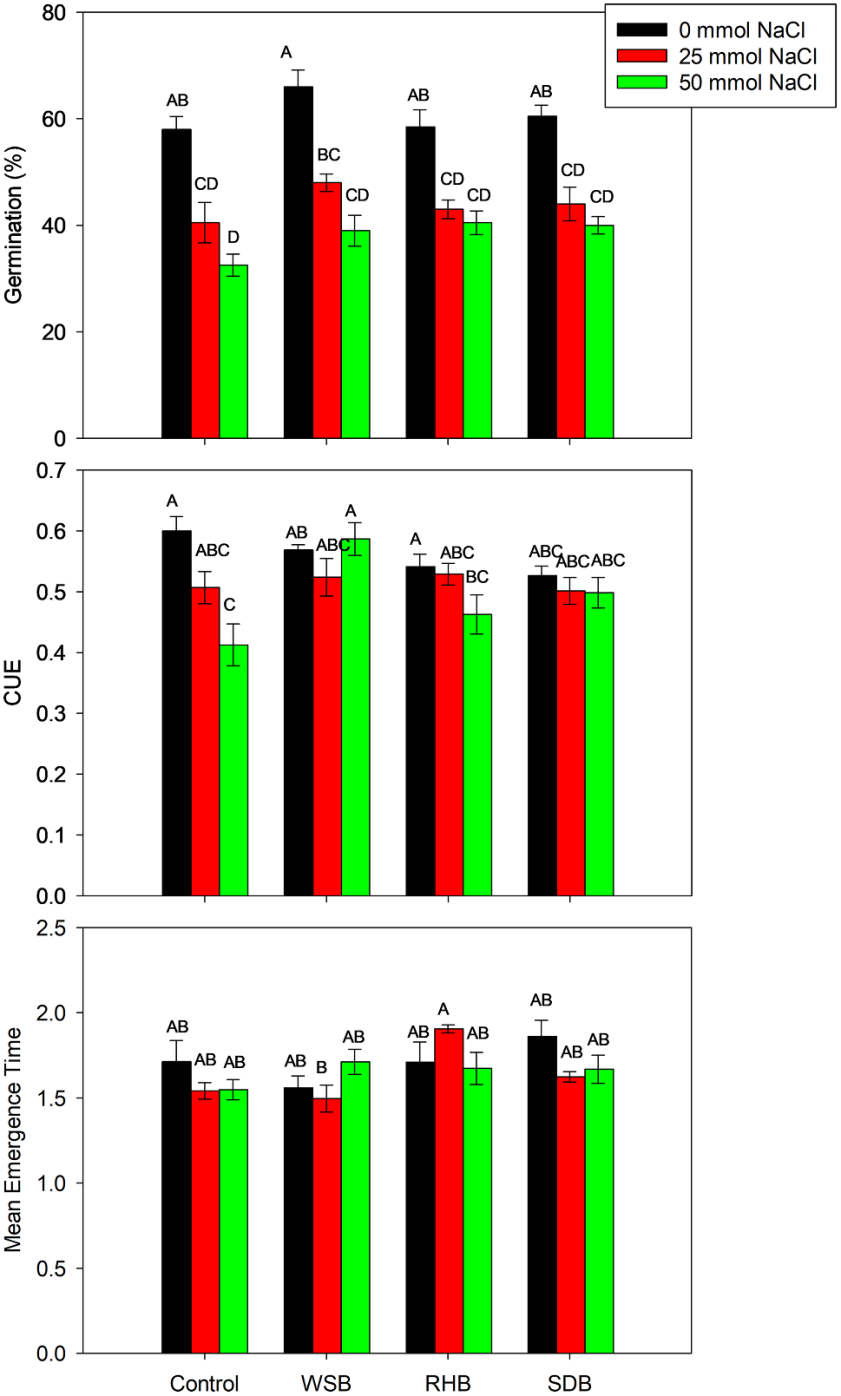
Interactive effects of biochar treatments (control, wheat straw biochar, rice husk biochar, and sawdust biochar) and salinity levels (0 mmol NaCl, 25 mmol NaCl, and 50 mmol NaCl) on germination parameters. The bars represent means ± standard error of four replicates. The letters above bars represent significant differences among treatments at three levels of salinity

The interactive effect of different salinity levels and biochar treatments on germination parameters is given in Fig. 1. The results show no significant interaction between salinity and biochar treatments on seed germination (Table 1). However, the main effects of salinity and biochars were statistically significant. The seed germination percentages varied among treatments and salinity levels. Under 0 mmol NaCl, control (no BC) and biochar treatments had the highest seed germination rate and were statistically at par with each other. Increasing NaCl stress decreased the germination percentage. The highest decrease was observed at 50 mmol NaCl. However, with BC application, the decline in germination was lower than control (no BC) (Fig. 1).

The coefficient of uniformity of emergence (CUE) provides insights into the evenness of seedling emergence due to different salinity levels and biochar treatments. At 0 mmol NaCl, Control had the highest CUE (0.60), followed by WSB at 50 mmol NaCl (0.59). Both treatments were statistically similar (Fig. 1). The lowest value of CUE was observed in the control (no biochar) at 50 mmol NaCl treatment.

The mean emergence time (MET) reflects the average time seedlings emerge. Our results show that at 25 mmol NaCl, RHB had the highest MET (1.90). Conversely, WSB at mmol NaCl resulted in the lowest MET (1.56). Despite these variations, the MET values were statistically similar across all treatments and salinity levels (Fig. 1).

### Effects of BCs on growth parameters of wheat under salt stress

The results for agronomic parameters of wheat in the present study was influenced by different salinity levels and biochar treatments; these data are shown in Table 2: There was no significant interaction between salinity and biochar treatments (Table 1) for the grain yield. The main effects of salinity and biochar treatments show that WSB and RHB exhibited higher grain yields (17% higher) than the control group. The treatment SDB also showed relatively higher grain yields over control. As the concentration of NaCl increased, grain yield decreased across all treatments, following a decreasing trend. The lowest values of grain yield were found at 50 mmol NaCl, followed by 25 mmol NaCl as compared to control.

The results for straw yield followed the same pattern as the grain yield (no significant interaction between biochar treatments and salinity levels). All three biochar treatments (WSB, RHB, and SDB) increased straw yield compared to the control treatment in a statistically similar trend. However, straw yield decreased across all treatments as salinity levels increased, following a decreasing trend.

Thousand grain weight is also an important indicator of grain quality and can reflect the impact of salinity and treatments on individual grain size. The results for 1000 grain weight (main effects) show that WSB treatment exhibited the highest 1000 grain weight (36.8 g), statistically higher than RHB and SDB, which caused 34.3 and 33.6 g, respectively. The main effects of salinity show that as salinity levels increased, 1000 grain weight decreased across all treatments, following a decreasing trend.

There was no significant interaction between biochar treatments and salinity levels for the number of tillers. The main effects show that biochar treatments increased the number of tillers but were statistically similar. Though the salinity levels decreased the number of tillers compared to the control, their effect was statistically identical.

The influence of biochar treatments and salinity levels on plant height showed a significant interaction. When there was no salinity (0 mmol NaCl), the plant height of WSB was the greatest, measuring 31.025. As salinity levels increased, plant height was generally decreased across all treatments, following a consistent declining trend. Even at 25 mmol NaCl, WSB still had the tallest plants, although this difference was not statistically significant compared to the other treatments. However, when the salinity level reached 50 mmol NaCl, all biochar treatments increased plant height, following a similar trend. Nevertheless, all treatments resulted in shorter plants compared to the no-salinity control.

**Table 2:** Interactive effects of biochar treatments (control, wheat straw biochar, rice husk biochar, and sawdust biochar) and salinity levels (0 mmol NaCl, 25 mmol NaCl, and 50 mmol NaCl) on agronomic parameters of plants in the pot experiment. The values are means ± standard error of four replicates. The letters in parentheses represent significant differences among treatments at three levels of salinity

		0 mM NaCl	25 mM NaCl	50 mM NaCl	Main effects
Grain Yield (g pot^− 1^)	Control	20.50 ± 0.96	18.25 ± 0.25	17.00 ± 0.58	18.58 (B)
	WSB	23.20 ± 0.82	19.50 ± 0.50	18.75 ± 0.48	20.48 (A)
	RHB	23.00 ± 0.58	19.75 ± 0.25	18.25 ± 0.25	20.33 (A)
	SDB	22.00 ± 0.82	19.00 ± 0.41	17.75 ± 0.25	19.58 (AB)
	Main effects	22.18 (A)	19.13 (B)	17.94 (C)	
Straw Yield (g pot^− 1^)	Control	18.50 ± 0.50	13.25 ± 0.48	12.50 ± 0.29	14.75 (B)
	WSB	21.00 ± 0.82	16.00 ± 0.82	14.50 ± 0.65	17.17 (A)
	RHB	20.50 ± 0.65	16.00 ± 0.41	14.00 ± 0.41	16.83 (A)
	SDB	19.75 ± 0.85	15.25 ± 0.48	14.00 ± 0.41	16.33 (A)
	Main effects	19.94 (A)	15.13 (B)	13.75 (C)	
1000 grain weight (G)	Control	36.41 ± 1.10	32.73 ± 0.56	29.52 ± 0.46	32.89 (C)
	WSB	41.26 ± 1.06	36.84 ± 0.27	32.34 ± 0.55	36.81 (A)
	RHB	38.43 ± 0.49	34.38 ± 0.35	30.26 ± 0.47	34.36 (B)
	SDB	37.72 ± 0.38	33.09 ± 0.83	30.12 ± 0.38	33.64 (BC)
	Main effects	38.46 (A)	34.26 (B)	30.56 (C)	
Plant Height (cm)	Control	28.68 ± 0.61 (B)	26.88 ± 0.19 (CDE)	24.55 ± 0.55 (F)	
	WSB	30.54 ± 0.51 (A)	27.55 ± 0.14 (CD)	26.40 ± 0.27 (DE)	
	RHB	29.38 ± 0.24 (B)	27.23 ± 0.16 (C)	26.55 ± 0.35 (DE)	
	SDB	28.73 ± 0.50 (B)	27.25 ± 0.68 (CD)	26.23 ± 0.20 (E)	
Number of tillers	Control	4.50 ± 0.29	3.50 ± 0.29	3.25 ± 0.25	3.75 (B)
	WSB	5.50 ± 0.29	4.25 ± 0.25	3.75 ± 0.25	4.50 (A)
	RHB	5.25 ± 0.25	4.00 ± 0.00	3.75 ± 0.25	4.33 (A)
	SDB	4.75 ± 0.25	4.00 ± 0.00	3.75 ± 0.25	4.17 (AB)
	Main effects	5.00 (A)	3.94 (B)	3.63 (B)	

### Sodium and K concentrations in wheat grains and straw

**Fig. 2:**
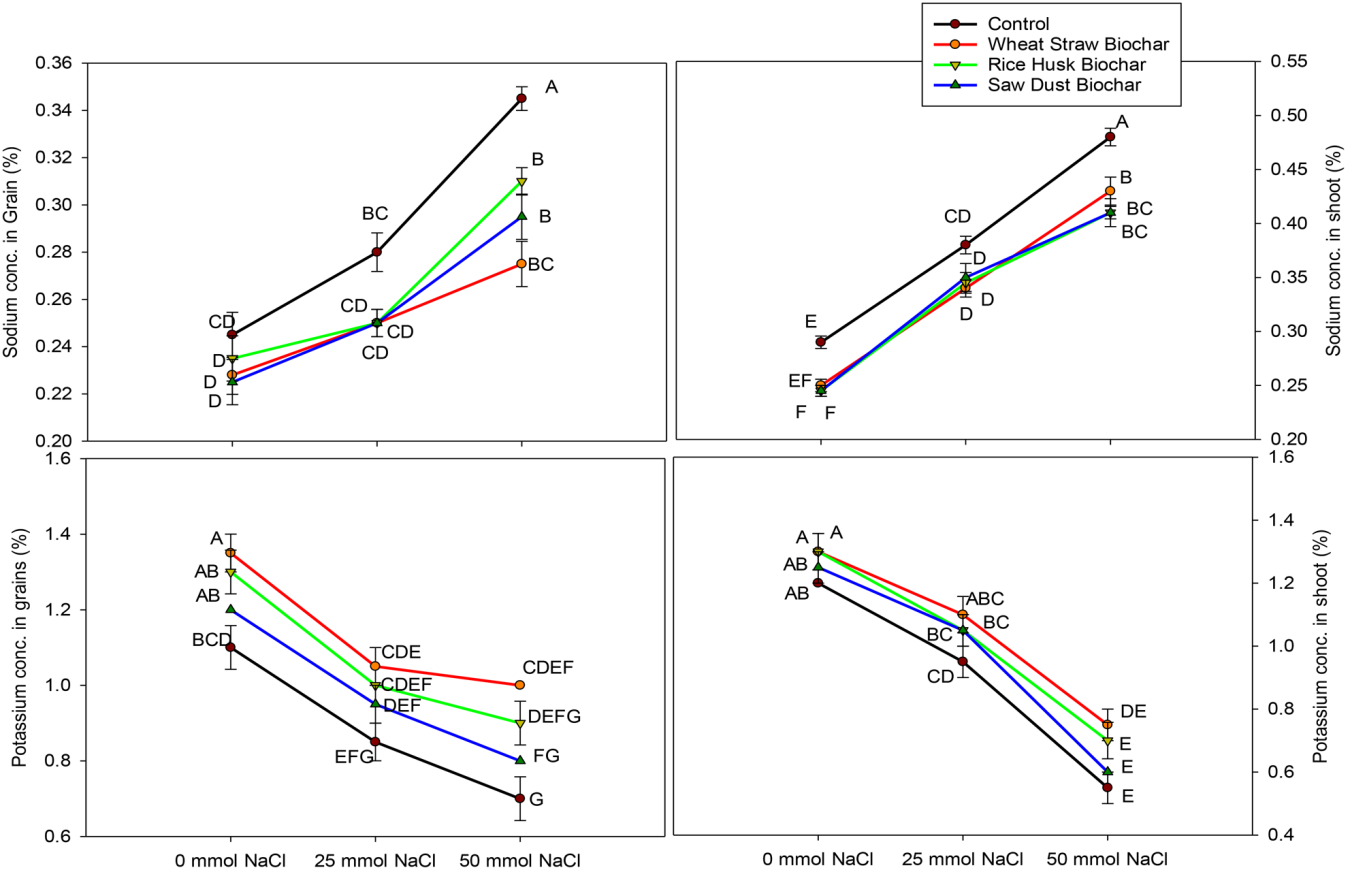
Interactive effects of biochar treatments (control, wheat straw biochar, rice husk biochar, and sawdust biochar) and salinity levels (0 mmol NaCl, 25 mmol NaCl, and 50 mmol NaCl) on concentration of potassium and sodium in plant shoots and grains. The data represent means ± standard error of four replicates. The letters above data points represent significant differences among treatments at three levels of salinity

The potassium (K) concentration in wheat grain and straw sample plants is provided in Fig. 2. At 0 mmol NaCl, WSB had the highest K concentration in wheat grains (1.35%), statistically like RHB and SDB. As salinity levels increased, K concentrations in wheat grains decreased across all treatments, with control (no BC) at 50 mmol NaCl exhibiting the lowest K concentration. The data regarding K concentration in straw samples shows that at 0 mmol NaCl, all BC treatments exhibited the highest K concentration in plant tissues compared to control, irrespective of NaCl application. As salinity levels increased, K concentrations in plant tissues generally decreased across all treatments. Interestingly, the RHB and SDB decreased K concentrations in wheat straw 0 mmol NaCl compared to the control (no BC).

Sodium (Na) concentration in wheat grains is an essential indicator of the quality and salt stress. In contrast, the Na concentration in straw reflects the ability of plants to exclude or tolerate high amounts of Na. Our results show that at 50 mmol NaCl, control (no BC) had the highest Na concentration in wheat grains, while the lowest values were found in all BCs at both 0 mmol NaCl and 25 mmol NaCl applications. The data regarding Na concentration in wheat straw show that at 50 mmol NaCl, the control had the highest Na concentration (0.480). As NaCl levels increased, the Na concentrations in plant straw remained relatively high across all treatments. However, the plants grown in BC-amended soils showed lower Na concentration than the control (no BC) at all levels of NaCl application (Fig. 2).

### Effects of BCs on soil properties

**Fig. 3:**
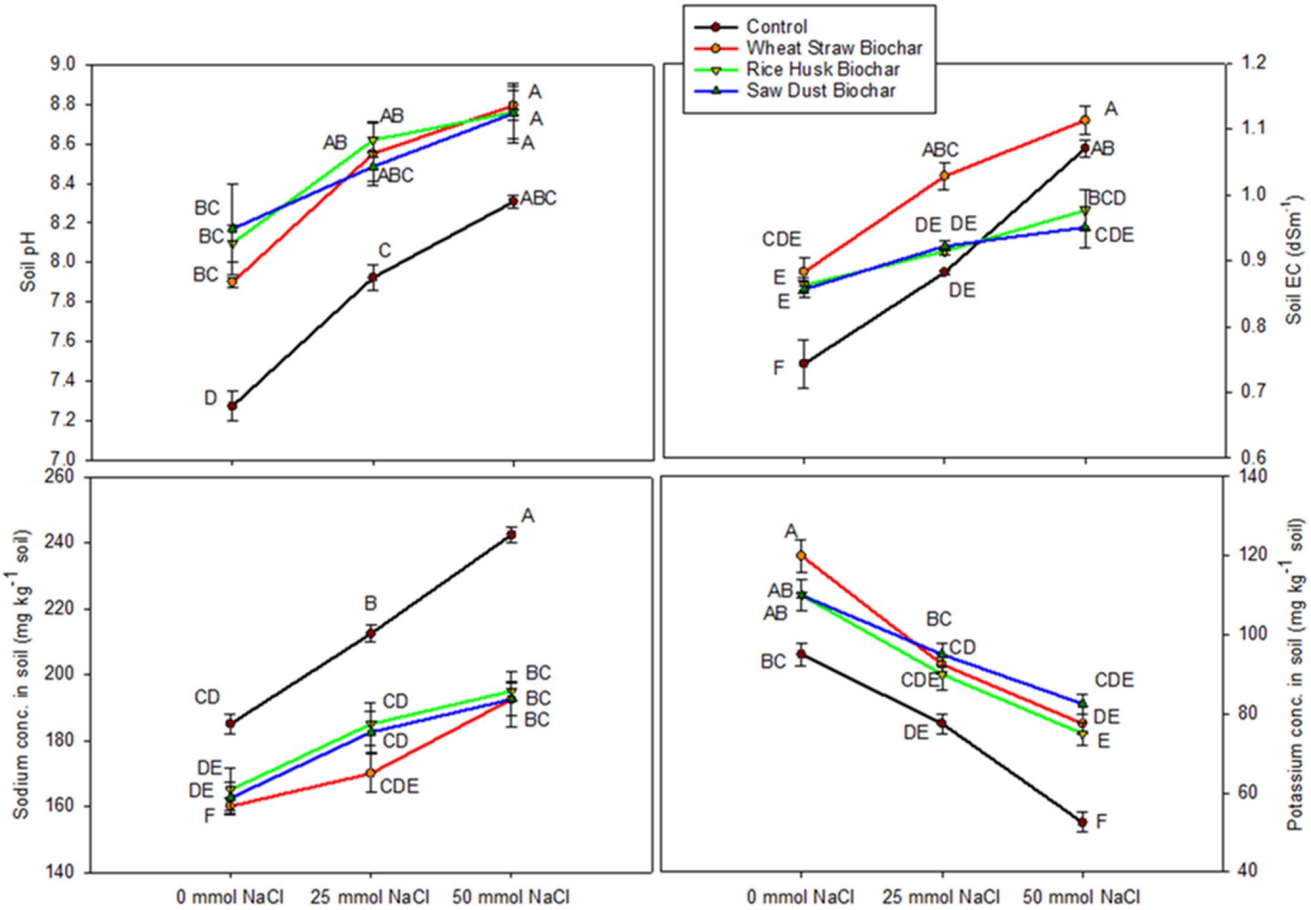
Interactive effects of biochar treatments (control, wheat straw biochar, rice husk biochar, and sawdust biochar) and salinity levels (0 mmol NaCl, 25 mmol NaCl, and 50 mmol NaCl) on soil pH, soil EC, and extractable concentrations of sodium and potassium in soil. The data represent means ± standard error of four replicates. The letters above data points represent significant differences among treatments at three levels of salinity

Soil pH is an essential factor that affects the availability of nutrients and the soil’s overall health. When the soil was exposed to 50 mmol NaCl, WSB showed the highest soil pH (8.79). RHB and SDB treatments also had similar high soil pH values at the same salinity level. These three treatments were statistically identical for their influence on soil pH. In contrast, control had the lowest soil pH (Fig. 3).

The results of soil EC show significant interaction between biochars and salinity levels for their effect (Table 1). As salinity levels increased, the soil EC generally increased across all treatments. At 50 mmol NaCl, WSB exhibited the highest values of EC. Control (no BC) at the same salinity level had the second-highest soil EC but was statistically like WSB. The RHB and SDB increased soil EC compared to control (0 mmol NaCl) but showed lower values within 50 mmol NaCl treatment.

In Fig. 3, the data shows the concentration of ammonium acetate extractable potassium (K). When no sodium chloride (NaCl) was added (0 mmol NaCl), the soil amended with WSB had the highest amount of potassium, measuring 120.00 mg kg^− 1^ soil. However, as the salinity levels increased, extractable K generally decreased for all treatments. This suggests that under salinity stress, the availability of K was reduced.

Ammonium acetate extractable sodium (Na) in the soil was at its maximum in the control group at 50 mmol NaCl. As salinity levels increased, the extractable Na content remained relatively high across all treatments. However, the concentration of Na in BC-amended soils was lower than in control soils (no biochar) at all NaCl levels (Fig. 3).

**Fig. 4:**
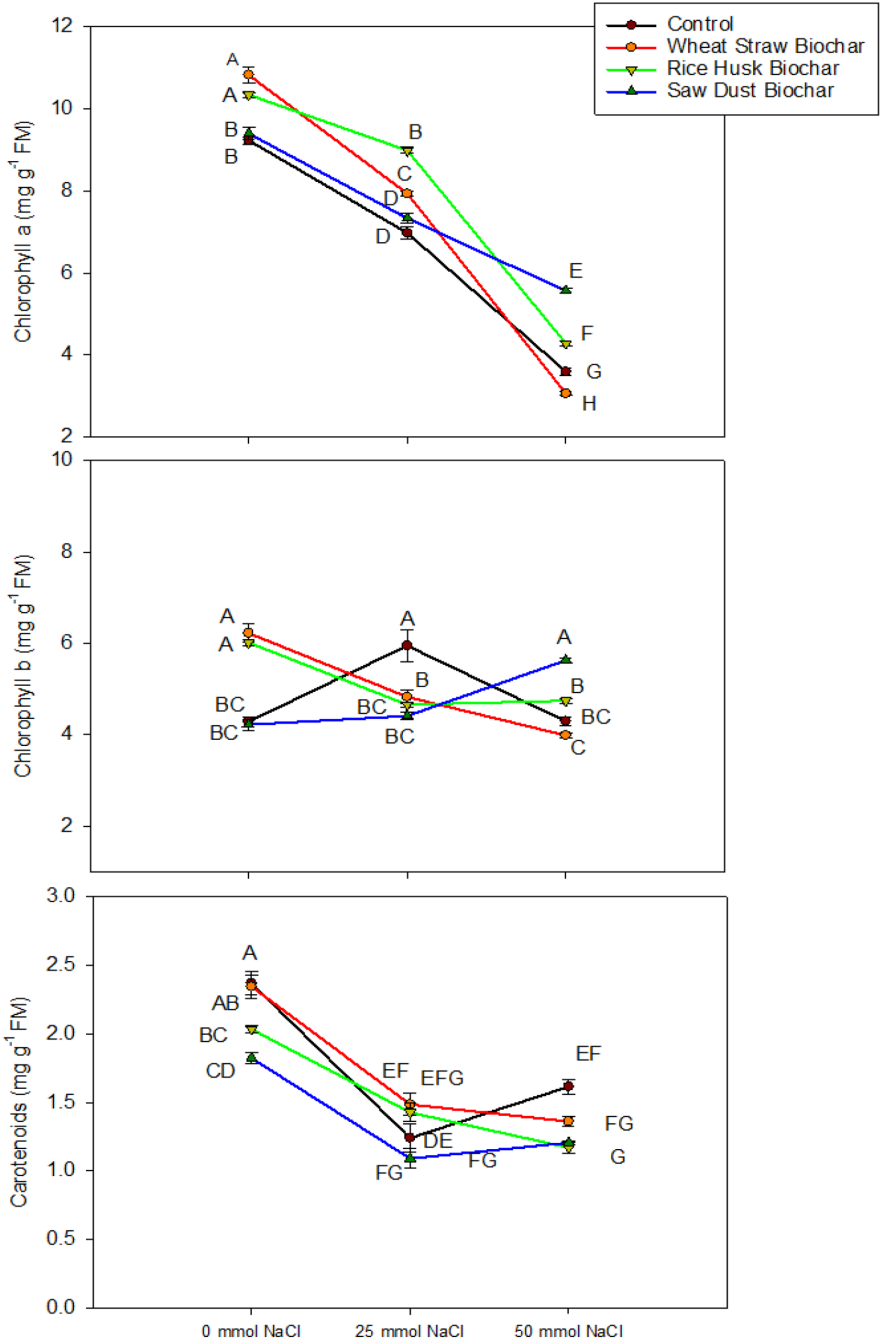
Interactive effects of biochar treatments (control, wheat straw biochar, rice husk biochar, and sawdust biochar) and salinity levels (0 mmol NaCl, 25 mmol NaCl, and 50 mmol NaCl) on chlorophyll a, b, and carotenoid concentration. The data represent means ± standard error of four replicates. The letters above data points represent significant differences among treatments at three levels of salinity

### Effects of BCs on photosynthetic and gas exchange attributes

The data regarding the interactive effects of biochar treatments at various NaCl levels on foliar chlorophyll a, b, and carotenoids were given in Fig. 4. There was a significant interaction between biochar treatments and salinity levels for the gas exchange parameters (Table 1). The foliar chlorophyll concentration exhibited substantial variations across different salinity levels and biochar treatments. In control (no BC), chlorophyll concentrations were lower across all salinity levels. The chlorophyll concentration decreased as the salinity level increased. However, with BC treatments, the decrease was more down than without BC. A slightly different trend was observed for chlorophyll b concentrations (Fig. 4). Under no salinity stress (0 mmol NaCl), the control had the lowest chlorophyll b concentration, which was statistically like the control (50 mmol NaCl) and SDB (at 0 and 25 mmol NaCl). As salinity increased to 25 mmol NaCl, chlorophyll b concentration in control increased significantly, statistically similar to WSB and RHB treatments at 0 mmol NaCl.

**Fig. 5:**
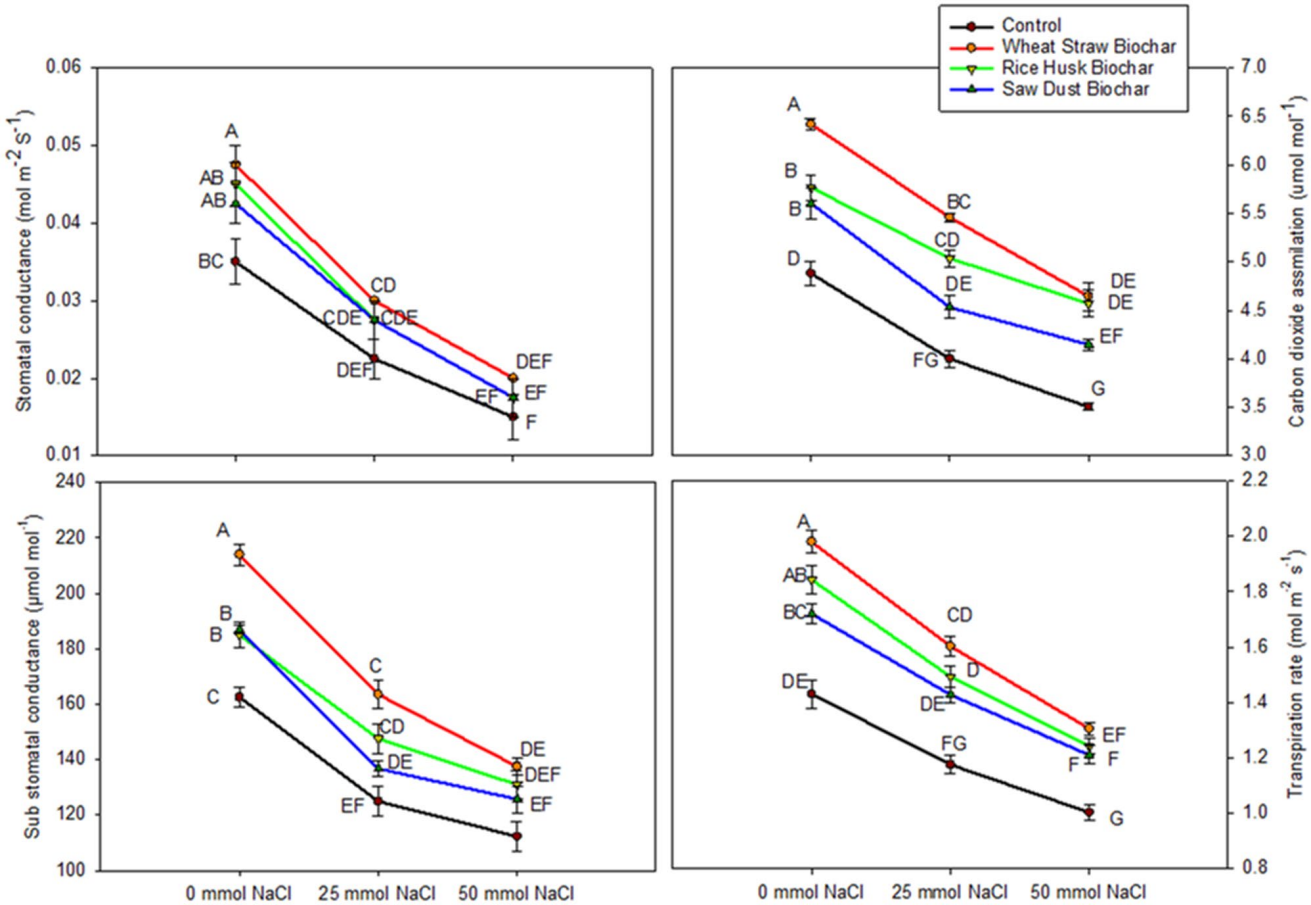
Interactive effects of biochar treatments (control, wheat straw biochar, rice husk biochar, and sawdust biochar) and salinity levels (0 mmol NaCl, 25 mmol NaCl, and 50 mmol NaCl) on gas exchange characteristics (gs (stomatal conductance, mol m^− 2^ s^− 1^), ci (sub stomatal CO_2_, µmol mol^− 1^), E (transpiration rate, mmol m^− 2^ s^− 1^) and A (CO_2_ assimilation rate (µmol m^− 2^ s^− 1^)) of wheat. The data represent means ± standard error of four replicates. The letters above different points represent significant differences among treatments at three levels of salinity

The carotenoid concentration results reveal interesting trends compared to the control (no BC) and the 0 mmol NaCl treatment. Without biochar amendments (Control), carotenoid concentrations were highest across all salinity levels. As salinity increased to 25 mmol NaCl, carotenoid concentration in the control decreased significantly. However, with BC treatments, the decrease was lower than without BC. The values for the gas exchange characteristics are given in the Fig. 5. The CO_2_ assimilation demonstrated distinct trends. Under no salinity stress (0 mmol NaCl), WSB had the highest assimilation rate. As salinity increased to 25 mmol NaCl, CO_2_ assimilation in WSB decreased significantly (15% compared to control). Though increasing NaCl stress decreased overall CO_2_ assimilation, in BC treatments, the decrease was lower than in control.

The stomatal conductance exhibited decreasing trends as salinity levels increased (Fig. 5). At 0 mmol NaCl, WSB had the highest conductance. As salinity increased to 25 mmol NaCl, stomatal conductance in WSB decreased significantly (36.8% decrease compared to the no-salinity control). The substomatal CO_2_ concentration showed decreasing trends with increasing salinity. At 0 mmol NaCl, WSB had the highest concentration, which dropped by 23% as the NaCl stress increased to 25 mmol NaCl. The lowest values were recorded at 50 mmol NaCl, where only WSB could cause a slight increase. The RHB and SDB were statistically similar with control at 50 mmol NaCl stress. The transpiration rate exhibited similar decreasing trends with increasing salinity. Moreover, among BCs, the WSB had the highest transpiration rate at 0 mmol NaCl, which fell as salinity increased to 25 mmol NaCl (Fig. 5).

### Correlation and principal component analysis

**Fig. 6:**
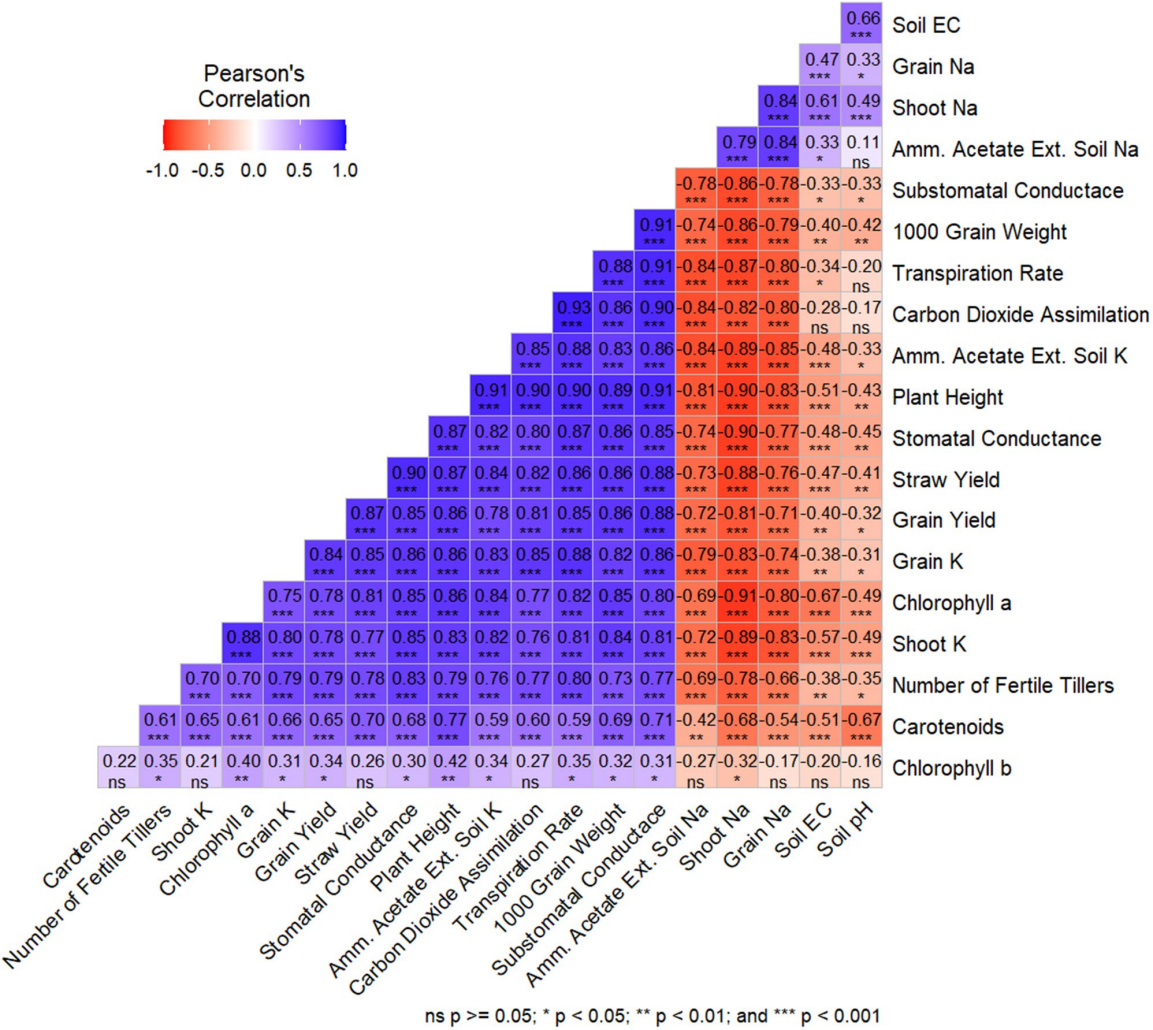
Pearson correlation between investigated parameters of the pot experiment

**Fig. 7:**
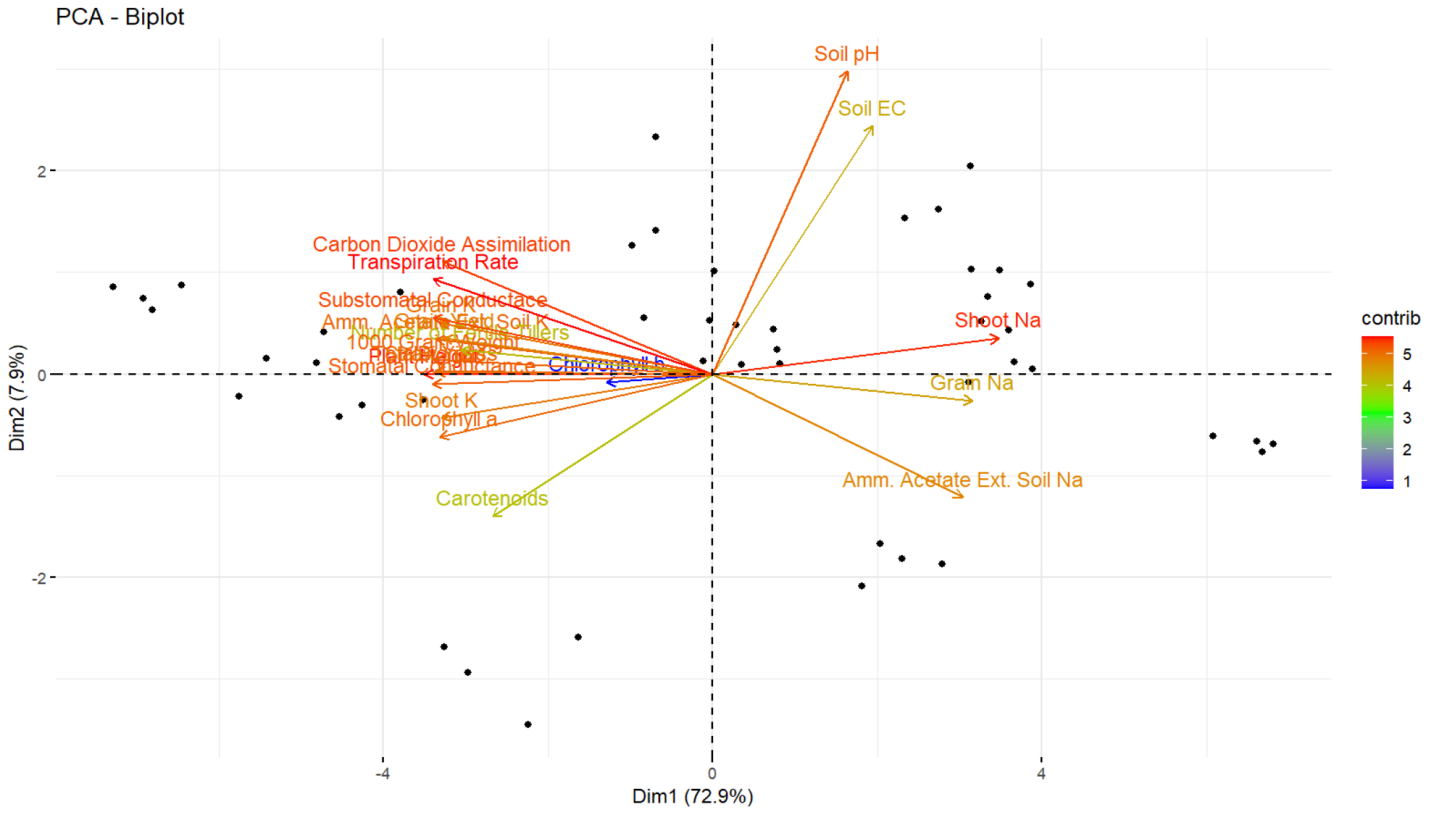
Principal component analysis of the investigated parameters

The Pearson correlation between studied variables is given in Fig. 6. The data showed a negative, highly significant correlation between soil Na concentration and all growth parameters. Soil Na concentration positively correlated with grain and shoot Na and soil EC. A similar pattern of negative correlation was found between shoot Na, grain Na, and other growth and physiological parameters. All remaining variables except Na, soil pH, and EC had a strong positive correlation. These results were further subjected to principal component analysis (Fig. 7), demonstrating that 97% and 0.15% of the total variance could be described in the first two components, respectively. In the first component, shoot K, chlorophyll a, all growth-related parameters, soil K, grain K, and gas exchange properties were positively correlated. In contrast, shoot Na, soil Na, and grain Na were negatively correlated. The second component negatively correlated soil pH, soil EC, and shoot Na. The remaining studied variables correlated in the third and onward components.

## Discussion

Salt stress affects seed germination by affecting osmotic potential and causing ion toxicity [35, 42]. The higher uniformity of emergence (CUE) in the control and the positive impact of wheat straw biochar (WSB) under moderate salinity conditions indicated that WSB can mitigate some salinity-induced stress during germination. Biochar has been reported to enhance seed germination under stress conditions [43] by improving soil aeration and water retention. The lower mean emergence time (MET) values with WSB at 0 mmol NaCl stress could be attributed to improved soil conditions due to BC application [30]. Applying BC to soils impacts physical properties and nutrient availability, improving seedling emergence and growth [37]. The variation in germination rates across BC treatments and salinity levels corroborates the findings of Zhang et al. [44], who observed that BC physicochemical properties can improve seed germination under stressed environments. The positive influence of WSB on seed germination rates under varying salinity levels could be due to its inherent characteristics that mitigate salinity effects, such as altering soil pH, improving nutrient availability, and reducing toxic ion contents [45].

The improvement in grain and straw yield, 1000-grain weight, tillers, and plant height with WSB application under varying salinity levels were related to the positive impact of BC on overall soil fertility and health. Biochar influences soil structure, nutrient cycling, and water-holding capacity, leading to improved crop growth and productivity [18, 46, 47]. The decrease in crop growth and yield attributes under increasing salt stress levels could be due to osmotic stress, ion toxicity, oxidative damage, and nutrient imbalance [8]. The ameliorating effects of WSB might be due to its ability in improving soil physical properties and nutrient uptake [48]. However, more research is required to determine the mechanisms behind this positive effect and optimal biochar application rates for different soil salinity levels [49].

This study demonstrated that salinity lowered the levels of photosynthetic pigments, i.e., foliar chlorophyll-a, and b. With increasing salinity, chlorophyll concentrations were substantially reduced (chlorophyll a by 24.3% and chlorophyll b by 7.2% at 25 mmol NaCl) compared to control. This could be due to salt stress-induced physiological disruptions, where toxic ions interfered with chloroplasts’ functioning. Several studies suggest that oxidative stress and nutrient imbalance under salt stress adversely affect chlorophyll biosynthesis [5, 50, 51]. Salt stress also significantly decreased the carotenoid contents. Biochar application (WSB and RHB) significantly increased carotenoid contents at 25 mmol NaCl. However, BC treatments did not substantially impact carotenoids at 50 mmol NaCl compared to the control. Results also showed a significant decrease in CO_2_ assimilation with increasing salt stress, which correlates well with reducing chlorophyll concentrations. Among biochars, the WSB exhibited the highest CO_2_ assimilation rate but showed a drop of 15% at 25 mmol NaCl compared to the control. The substomatal CO_2_ concentration under salinity stress also reflects a similar decline. In general, the findings of this study demonstrated the sensitivity of physiological parameters, including foliar chlorophyll content, CO_2_ assimilation, stomatal conductance, substomatal CO_2_ concentration, and transpiration rate, to salt stress [5, 9]. Many studies demonstrated an impaired carotenoid synthesis under salt stress and the positive role of biochar in ameliorating its negative impacts [52].

Furthermore, increasing salt stress reduced stomatal conductance, while WSB application improved conductance compared to control. Salinity-induced reduction in water loss through transpiration and reduced stomatal opening suggests its plant adaptation mechanism under stress conditions. Salt stress leads to stomatal closure to conserve water, altering photosynthetic efficiency [53, 54] and a recent study discussed the genetic basis of plants’ adaptive mechanisms to decrease water loss under salt stress [55].

The increase in soil pH after adding biochars is consistent with the alkaline nature of biochars. Biochar is rich in alkaline minerals and can neutralize soil acidity [18, 56]. Careful management is needed to avoid excessive soil alkalization, adversely affecting soil microbial activity and nutrient availability [57]. The increase in soil EC with increased salinity levels results from accumulating soluble salts [8]. Biochar, including WSB, may contain high ash content and alkaline minerals that can interact and lower soil salinity [58]. Increasing soil salinity levels decrease extractable potassium (K) in the soil. However, higher K content in soils treated with WSB indicates that BC helped improve potassium availability in saline soils. Biochar can increase nutrient availability by influencing cation exchange capacity and organic matter content in soil [59].

Interestingly, the unchanged Na levels in soils treated with BC might suggest a different type of interaction between BC and Na in the soil. While BC can affect many soil characteristics, its impact on soil Na levels under saline conditions may be less pronounced. This could be due to the high mobility and solubility of Na ions in the soil [60].

The improved K concentration in plant tissues in the WSB treatment under salt stress indicated higher K availability due to BC, which ameliorated the ion imbalance [28]. Specifically, the addition of BCs improved not only the soil structure; but plausibly, influencing the nutrient dynamics in soil (decreased Na bioavailability and vice versa for K) [61], leading to improved grain quality under saline conditions [62]. However, its impact on plant tissue Na levels is limited, suggesting more research is needed to understand the mechanisms of BC interactions within the soil and plant systems under salt stress conditions [48].

## Conclusion

The present experiment revealed that wheat straw biochar was more effective than other biochar types in mitigating the negative effects of salinity on seed germination and early growth of wheat by improving soil properties such as soil pH, EC, and nutrient status. Moreover, the results indicated that biochar application was proven effective in improving wheat growth and productivity seedlings growth under saline conditions. Specifically, the biochar amendements might have modulated sodium concentration in plants and ultimately the grains; further research is needed to understand the precise mechanisms in wheat growing in different soil types and salinity under field conditions. Moreover, long-term experimentation invovling biochar under different soil types and research directions (e.g., exploring the microbial communities associated with biochar application) is needed in future studies.

### Electronic supplementary material

Below is the link to the electronic supplementary material.

Supplementary Material 1

## Data Availability

All data supporting the findings of this study are available within the paper.
