# Supplementary material for: Effects of biochar types on seed germination, growth, chlorophyll contents, grain yield, sodium, and potassium uptake by wheat (Triticum aestivum L.) under salt stress
Source: BMC Plant Biol. 2024 Jun 1;24:487. doi: 10.1186/s12870-024-05188-0 (PMC11143699; doi:10.1186/s12870-024-05188-0)
Supplement: Supplementary file 1 — Supplementary Material 1 [file 12870_2024_5188_MOESM1_ESM.docx]

**Effect of biochar types on seed germination, growth, chlorophyll contents, grain yield, sodium, and potassium uptake by wheat (*Tritium aestivum* L.) under salt stress**

Sumei Duan^1^, Arwa Abdulkreem AL-Huqail^2^, Ibtisam Mohammed Alsudays^3^, Mobeen Younas^4^, Alishba Aslam^4^, Ahmad Naeem Shahzad^5^, Muhammad Farooq Qayyum^4^*, Muhammad Rizwan^6^, Yousef Alhaj Hamoud^7^, Hiba Shaghaleh^8^, Jean Wan Hong Yong^9^**

*^1^ College of Agriculture, Anhui Science and Technology University, Huainan City, Anhui, China*

*^2^ Department of Biology, College of Science, Princess Nourah bint Abdulrahman University, P.O.Box 84428, Riyadh 11671, Saudi Arabia*

*^3^ Department of Biology, College of Science, Qassim University, Burydah 52571, Saudi Arabia*

*^4^ Department of Soil Science, Faculty of Agricultural Sciences & Technology, Bahauddin Zakariya University Multan, Pakistan*

*^5^ Institute of Agronomy, Faculty of Agricultural Sciences & Technology, Bahauddin Zakariya University Multan, Pakistan*

*^6^ Department of Environmental Sciences, Government College University Faisalabad, Faisalabad, 38000, Pakistan*

*^7^ College of Hydrology and Water Resources, Hohai University, Nanjing 210098, China*

*^8^ College of Environment, Hohai University, Nanjing 210098, China*

*^9^ Department of Biosystems and Technology, Swedish University of Agricultural Sciences, 23456 Alnarp, Sweden*

***Corresponding author:** [farooq.qayyum@bzu.edu.pk](mailto:farooq.qayyum@bzu.edu.pk)

****Corresponding author:** [jean.yong@slu.se](mailto:jean.yong@slu.se)

**Table S1.** Physiochemical properties of the biochars used in seed germination and plant growth experiments.

| Biochars | N (%) | P (%) | Volatile matter (%) | Ash content (%) | EC_1:5_ (µs/m) | pH_1:5_ | BET surface area (m^2^ g^-1^) |
| --- | --- | --- | --- | --- | --- | --- | --- |
| Wheat-straw biochar | 0.012 | 0.08 | 35.5 | 33.7 | 536 | 8.5 | 91.0 |
| Rice-husk biochar | 0.016 | 0.089 | 26 | 31.8 | 231 | 7.61 | 75.0 |
| Saw-dust biochar | 0.003 | 0.12 | 37 | 30.22 | 340 | 8.0 | 94.0 |
